# Radioligand Assay-Based Detection of Antibodies against SARS-CoV-2 in Hospital Workers Treating Patients with Severe COVID-19 in Japan

**DOI:** 10.3390/v13020347

**Published:** 2021-02-23

**Authors:** Hidenori Matsunaga, Akiko Makino, Yasuhiro Kato, Teruaki Murakami, Yuta Yamaguchi, Atsushi Kumanogoh, Yuichiro Oba, Satoshi Fujimi, Tomoyuki Honda, Keizo Tomonaga

**Affiliations:** 1 Department of Psychiatry, Osaka General Medical Center, Osaka 558-8558, Japan; 2 Laboratory of RNA Viruses, Department of Virus Research, Institute for Frontier Life and Medical Sciences, Kyoto University, Kyoto 606-8507, Japan; makino@infront.kyoto-u.ac.jp (A.M.); tomonaga.keizo.5r@kyoto-u.ac.jp (K.T.); 3 Department of Respiratory Medicine and Clinical Immunology, Graduate School of Medicine, Osaka University, Osaka 565-0871, Japan; kato@imed3.med.osaka-u.ac.jp (Y.K.); t.murakami@imed3.med.osaka-u.ac.jp (T.M.); y.yamaguchi@imed3.med.osaka-u.ac.jp (Y.Y.); kumanogo@imed3.med.osaka-u.ac.jp (A.K.); 4 Department of General Medicine, Osaka General Medical Center, Osaka 558-8558, Japan; oobau@mbj.ocn.ne.jp; 5 Division of Trauma and Surgical Critical Care, Osaka General Medical Center, Osaka 558-8558, Japan; fujimis@opho.jp; 6 Division of Virology, Department of Microbiology and Immunology, Graduate School of Medicine, Osaka University, Osaka 565-0871, Japan; thonda@virus.med.osaka-u.ac.jp

**Keywords:** SARS-CoV-2, antibody, radioligand assay, hospital staff, low-titer, cross reaction

## Abstract

This study aimed to clarify whether infection by severe acute respiratory syndrome coronavirus 2 (SARS-CoV-2) is prevalent among the staff of a hospital providing treatment to patients with severe coronavirus disease 2019 (COVID-19) using radioligand assay (RLA). One thousand samples from the staff of a general hospital providing treatment to patients with severe COVID-19 were assayed for SARS-CoV-2 nucleocapsid protein (N) IgG using RLA. Nine patients with COVID-19 who had been treated in inpatient settings and had already recovered were used as control subjects, and 186 blood donor samples obtained more than 10 years ago were used as negative controls. Four of the 1000 samples showed apparently positive results, and approximately 10 or more samples showed slightly high counts. Interestingly, a few among the blood donor samples also showed slightly high values. To validate the results, antibody examinations using ELISA and neutralizing antibody tests were performed on 21 samples, and chemiluminescence immunoassay (CLIA) was performed on 201 samples, both resulting in a very high correlation. One blood donor sample showed slightly positive results in both RLA and CLIA, suggesting a cross-reaction. This study showed that five months after the pandemic began in Japan, the staff of a general hospital with a tertiary emergency medical facility had an extremely low seroprevalence of the antibodies against SARS-CoV-2. Further investigation will be needed to determine whether the slightly high results were due to cross-reactions or a low titer of anti-SARS-CoV-2 antibodies. The quantitative RLA was considered sensitive enough to detect low titers of antibodies.

## 1. Introduction

The outbreak of severe acute respiratory syndrome, caused by severe acute respiratory syndrome coronavirus 2 (SARS-CoV-2), which was first recognized in December 2019, has resulted in a worldwide pandemic. As of November 2020, more than 63 million people have been infected, and approximately 1.5 million have died of the infection (COVID-19) (http://www.worldometers.info/coronavirus/#countries, accessed on 30 November 2020). In Japan, a large cluster of infections, in which more than 700 people were infected, occurred in a cruise ship in February. Thereafter, the first phase of the epidemic, in which more than 16,000 people were infected, occurred from March to May 2020. After several weeks, the second phase of the epidemic occurred, with a peak in the first half of August, in which more than 80,000 people were infected between July and the end of October (http://hazard.yahoo.co.jp/article/20200207, accessed on 30 November 2020). And then, the third phase started in November. Osaka General Medical Center (OGMC), with a tertiary emergency medical facility, has played a key role in treating patients with severe COVID-19 in Osaka Prefecture, and during each phase of the epidemic, more than a hundred patients with moderate to severe COVID-19 had been admitted. In this study, to confirm the effectiveness of infection protection among hospital workers, blood samples from one thousand hospital staff were collected in mid-July, just at the beginning of the second phase of the epidemic in Japan. The levels of anti-SARS-CoV-2 antibodies were measured using a radioligand assay (RLA) and the results were validated using other assays.

## 2. Materials and Methods

### 2.1. Subjects

Of the approximately 2000 employees, only 1000 participated in this study, including 171 doctors, 497 nurses, 198 other healthcare professionals, and 134 office workers (male/female ratio: 276/724; age: median 34 years, range 21–69 years). 

Nine patients with COVID-19, who had been treated in inpatient settings at the OGMC and had already recovered, were recruited as positive controls, and their blood samples were collected from 5th June to 7th July, approximately 22–89 (median 65) days after symptom onset. Six patients had been treated with respirators, and one of them had extracorporeal membrane oxygenation (ECMO). As negative controls, 186 blood donor samples, frozen for more than 10 years, were used. 

### 2.2. Ethics Approval

This study was approved by the Ethics Committee of OGMC, and written informed consent was obtained from all participants, including the patients with COVID-19.

### 2.3. Radioligand Assay (RLA)

**Figure 1:**
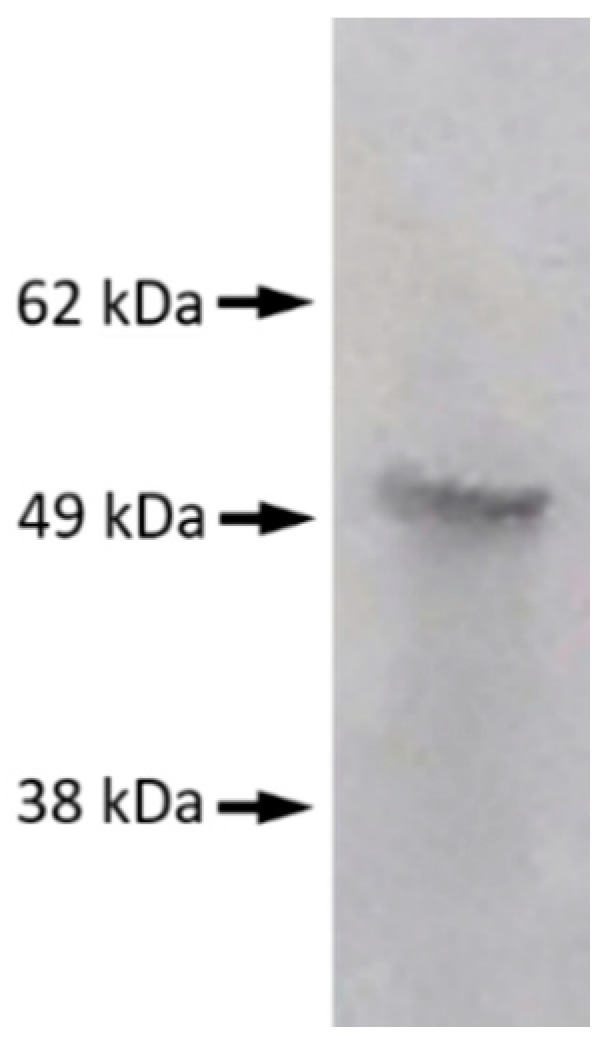
Verification of the severe acute respiratory syndrome coronavirus 2 (SARS-CoV-2) N protein using electrophoresis. A single band of approximately 50 kDa was recognized for SARS-CoV-2 N.

RLA was used to detect the antibody against the nucleocapsid protein (N) of SARS-CoV-2 (the antigen) [1,2]. cDNA encoding SARS-CoV-2 N with a His-tag and a T7-tag at the N-terminal was inserted into pET28a. Since N contains 7 methionine residues and T7-tag contains 3, a total of 10 35S-methionine per molecule could play the role of a marker for the protein. In-vitro transcription and translation were conducted using a reticulocyte lysate kit (TNT Quick Coupled Transcription/Translation SystemTM, Promega, Madison, WI, USA) and 35-S-methionine (Perkin Elmer, Waltham, MA, USA) by incubating at 30 °C for 90 min and radiolabeled N protein was separated from that with unincorporated 35S-methionine using a column (Nick ColumnTM, Cytiva, Marlborough, MA, USA). Products were analyzed by SDS-PAGE and autoradiographed to demonstrate the presence of SARS-CoV-2 N (Figure 1). Tris-buffered saline with 0.1% bovine serum albumin and 0.1% Tween 20 was used for the antigen-antibody reaction. Ninety-six well-containing filter plates (MultiScreenTM, Merk, Darmstadt, Germany) were used as containers of a 50-µL reaction mixture, including 1 µL of serum. Resin adsorbing human IgG (Protein G Sepharose 4 Fast FlowTM, Cytiva, Marlborough, MA, USA) was added to precipitate the antigen-antibody complex. After incubation, the precipitate was washed with Tris-buffered saline containing 1% Tween 20 four times in one hour by aspirating the buffer through the filter at the bottom of the wells. After drying, the scintillation cocktail (Optiphase SuperMixTM, Perkin Elmer Life Science, Boston, MA, USA) was added, and radioactivities were counted. 

Three samples with different antibody titers from patients with COVID-19 were used as positive controls. To reduce the difference between assays, the index value was used; the average of the negative samples in the plate, excluding those with high or slightly high values, was considered to have an index value of 0, and the average from three positive controls was determined to have an index value of 8. The mean (standard deviation) index value of negative samples within each plate became 0 (0.2–0.3), and the mean (standard deviation) index value of the three positive samples in all 13 plates tested was 10.13 (0.57), 8.33 (0.03), and 5.63 (0.54), respectively.

### 2.4. Enzyme-Linked Immunosorbent Assays (ELISA)

To validate the RLA results, ELISA and neutralizing antibody titration were performed for 21 selected samples, including nine from patients with COVID-19, and four apparently positive, six slightly high, and two negative samples from hospital staff. Anti-SARS-CoV-2 N IgG and anti-SARS-CoV-2 S IgG in the sera were measured using Novel Coronavirus COVID-19 IgG ELISA TM(DRG International, Inc., Springfield, NJ, USA) and COVID-19 Human IgM IgG ELISA kit (Spike protein)TM (CELLSPECT, Morioka, Japan), according to the manufacturer’s protocol.

### 2.5. Neutralization Assay

One hundred TCID50 of SARS-CoV-2/ Japan/UT-NCGM02 was reacted with 2-fold serial diluted serum at 37 °C for 1 h. The mixture was overlaid onto Vero E6/TMPRSS2 cells for 1 h. After washing, the inoculated cells were cultured in Dulbecco’s Modified Eagle’s Medium (Thermo Fisher Scientific, Waltham, MA, USA) with 2% FCS. Three days post-infection, the neutralizing antibody titer was determined by observation of the cytopathic effect.

### 2.6. Chemiluminescence Immunoassay (CLIA)

To further validate the RLA results, CLIA measurements using an automatic analyzer were also performed because it had been validated by other researchers [3,4] and was available for research use. For CLIA, 201 samples were selected, including nine samples from patients with COVID-19, all 22 samples from the hospital staff having 1.0 or more index values by RLA, 169 negative samples having less than 1.0 index values by RIA from the hospital staff, and one blood donor sample with slightly high counts. CLIA was performed using a fully automatic analyzer (iFlashTM, YHLO Biotechnology Company Ltd., Shenzhen, China). In this assay system, magnetic beads coated with SARS-CoV-2 N and S were used as the antigens.

### 2.7. Statistical Analysis 

To compare the correlation between the results of the two methods, Spearman’s rank correlation coefficient was used.

## 3. Results

### 3.1. Verification of RLA

**Figure 2:**
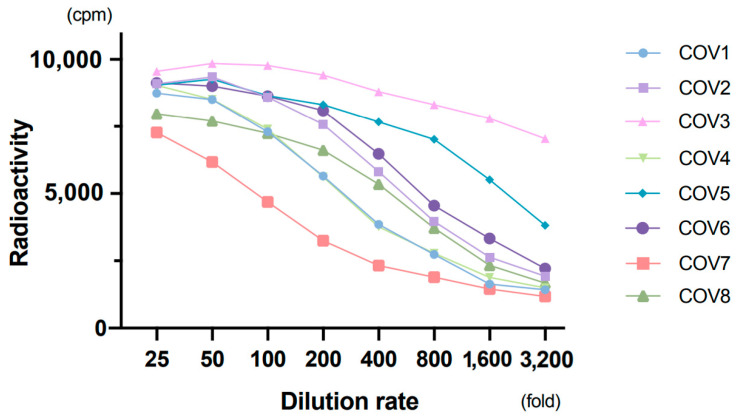
Dilution test. Eight samples from patients with coronavirus disease 2019 (COVID-19) were used with two-fold serial dilution.

**Figure 3:**
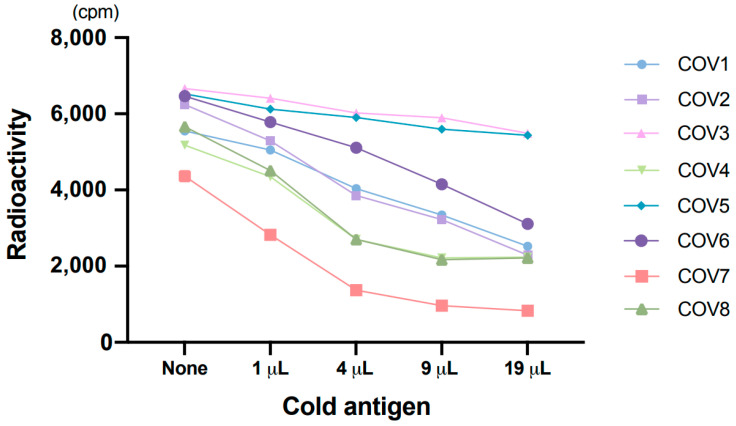
Absorption test. Non-radiolabeled (cold) antigens were used to absorb specific antibodies.

All 9 patients with COVID-19 showed apparently high counts by RLA, which means a high titer of antibodies. Dilution tests in eight patients showed a gradual decrease in radioactivity with increasing dilution (Figure 2). We also performed the absorption test in eight samples from patients with COVID-19 to verify the test performance. One micro-liter of serum was mixed with 0, 1, 4, 9, and 19 µL of non-radiolabeled antigens, produced the same way as radiolabeled antigens, and reaction buffer was added to make a total volume of 30 µL; the specific antibodies were allowed to absorb the non-radiolabeled antigens. After 1 h of incubation at 4 °C, the normal assay procedure with radiolabeled antigens, equivalent amount of 1 µL of cold antigens per well, was performed. Rates of decrease in radioactivity were greater when a higher amount of non-radiolabeled antigens were used for pretreatment (Figure 3). These findings indicated that anti-SARS-CoV-2 N IgG was reliably detected by RLA. 

### 3.2. RLA Results

**Figure 4:**
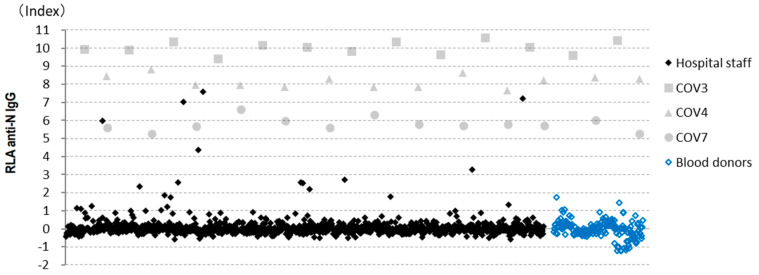
Radioligand assay (RLA) results. Results obtained from all 13 assays were collated, including those from the hospital staff (*n* = 1000) and blood donors (*n* = 186). COV3, 4, and 7 were the positive controls.

RLA was performed on samples from 1000 hospital staff and 186 blood donors using 13 96-well filter plates. Although the results for the highest positive control were 6000–7000 cpm and those for the negative samples were approximately 1000 cpm, the standard deviation across negative samples was very small; therefore, the samples with relatively high counts could be clearly discriminated from negative samples. For example, in the first assay, the average and standard deviation of 89 samples, excluding those with high and slightly high counts, were 1083 cpm and 130 cpm, respectively. All the results are shown in Figure 4. The number and rates of positive samples for various cut-off values are shown in Table 1. 

### 3.3. ELISA and Neutralizing Antibody Titration Results

**Figure 5:**
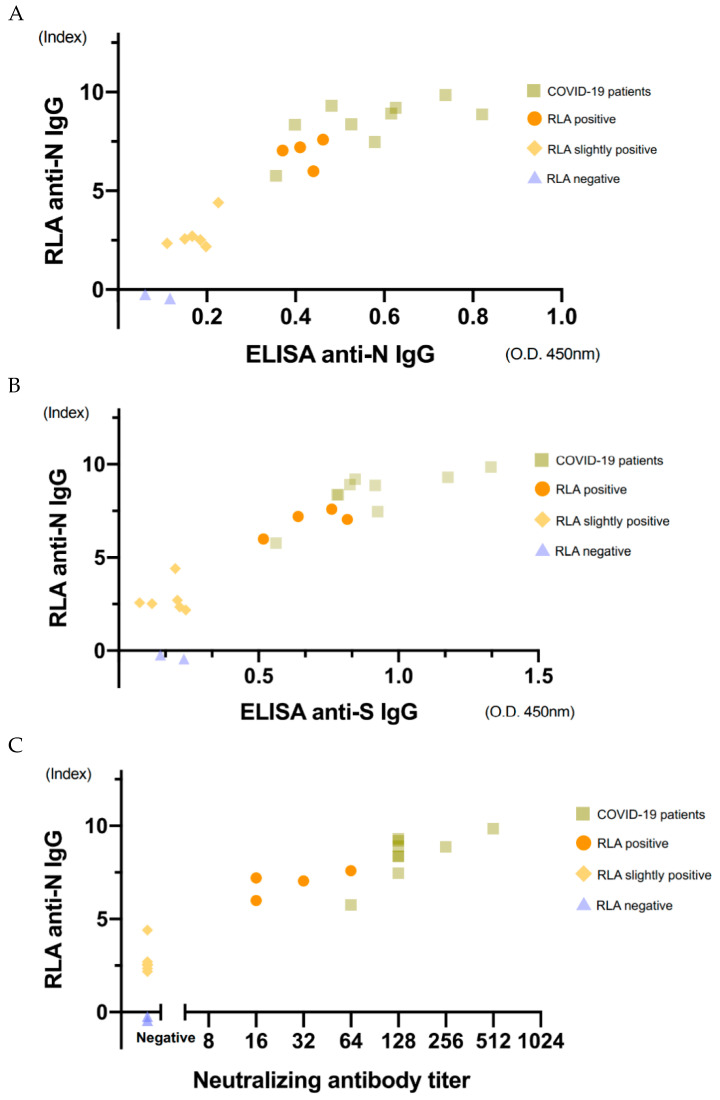
Correlation of the results obtained using RLA with those obtained using ELISA and neutralization titers. The 21 selected samples consisted of nine samples from patients with COVID-19 and four apparently positive, six slightly high, and two negative samples from hospital staff. Correlation of the results obtained using RLA with anti-SARS-CoV-2 N determined using ELISA (**A**), anti-SARS-CoV-2 S determined using ELISA (**B**), and neutralization antibody titers (**C**). The correlation coefficients were 0.92, 0.87, 0.93 (Spearman’s rank correlation coefficient), respectively.

To verify the RLA results, measurements using ELISA and neutralizing antibody titration were performed for some selected 21 samples. Correlations with the RLA results are shown in Figure 5. The values of anti-N IgG and anti-S IgG by ELISA and neutralizing antibody titration were well correlated with those of anti-N IgG by RLA (rs = 0.92, 0.87, 0.93, respectively; Spearman’s rank correlation coefficient).

Nine COVID-19 samples and four positive samples from hospital staff showed anti-N IgG and anti-S IgG, according to ELISA and the neutralizing antibody test. However, six samples with slightly high counts in RLA showed slightly high values for anti-N IgG, but not for anti-S IgG, according to ELISA and the neutralizing antibody test.

### 3.4. CLIA Results, Including IgM Results

**Figure 6:**
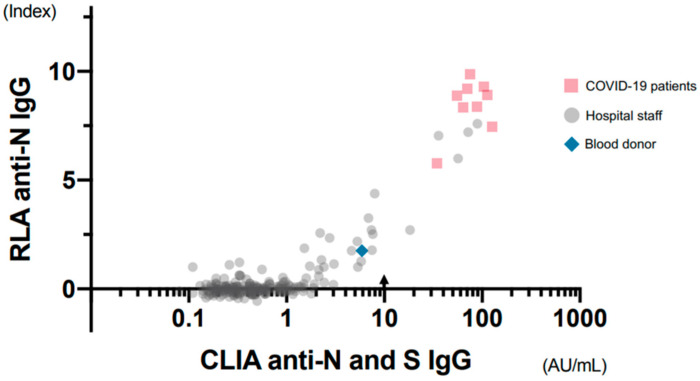
Correlation of the results obtained using RLA and CLIA. The 201 selected samples consisted of nine samples from patients with COVID-19, all 24 samples from the hospital staff having 1.0 or more index values by RLA, 167 negative samples from the hospital staff, and one blood donor sample with slightly high counts. The correlation coefficient was 0.92 among the 52 samples, excluding negative ones whose results were lower than 1.0 in CLIA (Spearman’s rank correlation coefficient). Arrow: Cut-off point recommended by manufacturers.

CLIA measurements were performed on 201 samples, including those from 9 patients with COVID-19, 191 hospital staff, and one blood donor with slightly high counts. Among the samples from hospital staff, 809 out of 976 samples with index values lower than 1.0 were excluded (Figure 6). A cut-off value of 10.0 AU/mL was recommended for CLIA by the manufacturer.

Among the 52 samples, excluding 149 negative ones whose results were lower than 1.0 in CLIA, a good correlation was observed between the results of CLIA and RLA (rs = 0.92, Spearman’s rank correlation coefficient). One blood donor sample with an index value of 1.75 in RLA showed a slightly high value of 5.9 AU/mL in CLIA, although it was below the cut-off point. Anti-SARS-CoV-2 IgM was also measured by CLIA, and only three samples showed specific IgM, all from patients with COVID-19. Each sample was taken on the 22nd, 51st, and 68th days from the onset of symptoms. Two of the patients had recovered from serious conditions, requiring artificial respiration.

## 4. Discussion

Various commercialized kits have been developed to date for testing anti-SARS-CoV-2 antibodies; however, we measured the anti-N IgG using RLA. RLA was first introduced in 1997 by Yamamoto et al. [5] to detect autoantibodies against cytochrome CYP2D6, which causes autoimmune hepatitis, more sensitively than immunoblotting and ELISA. In RLA, the antigen contains 35S-labeled methionine as a marker, and hence, no additional chemical agent is required for luminescence or color development. Radioactivity is a marker of the antigen, and even small amounts of it can be detected accurately. Moreover, the antigen can be mixed with serum in the liquid phase without immobilization. Since the antigen completely preserves the natural conformation, a conformational antibody can be detected. We had previously attempted to detect autoantibodies against neurotransmitter receptors [6], after which we used the same method to measure antibodies against Borna disease virus [1,2]. Since the Borna disease virus infects human nervous cells latently and persistently, the titers of specific antibodies were relatively low. Therefore, it is difficult to obtain consistent results using various antibody detection methods. In our experience, RLA seems to be quite sensitive, quantitative, and reproducible, and can potentially be used as an excellent tool for measuring antibodies precisely. 

Using RLA, all samples from patients with COVID-19 were found to show high counts. Both the dilution test and the absorption test showed reasonable results. The results of ELISA and neutralizing antibody titration were consistent with those of RLA. Moreover, CLIA, commercialized and validated by other researchers [3,4], showed consistency in results with RLA. Therefore, RLA was considered to detect anti-SARS-CoV-2 IgG reliably. 

Four out of 1000 samples from hospital staff showed positive counts as high as in patients with COVID-19. Three of them had symptoms and were subsequently diagnosed with COVID-19. The rest had not been diagnosed with COVID-19, although they had experienced a fever for a week. When we planned this study, we expected to find a mildly or symptom-free infected person based on the specific antibodies; however, we did not find such infected individuals. One reason for this might be the very low prevalence of the infection in July 2020 when the blood samples were collected, as no staff with subclinical COVID-19 could be included in this study. Another possibility is that such infected persons had very low titer of antibodies and, were thus included in the group with slightly high counts. 

Plebani et al. [7] surveyed SARS-CoV-2 infection across 8285 health care workers whose blood samples were collected between 22nd February and 29th May; their anti-N and anti-S IgG and IgM levels were measured subsequently. Among them, 378 (4.6%) were seropositive and 286 (3.5%) were positive for viral genomes. Narrowing in on 286 PCR-positive cohorts, 210 (73%) were found seropositive. The seropositivities differed according to the severity of symptoms (severe and hospitalized, mild disease, and asymptomatic were 100%, 83%, and 58%, respectively). In the United States, CDC had examined seroprevalence of anti-SARS-CoV-2 S antibodies in 3248 health professionals, sampled between 3rd April and 19th June [8]. Among 194 (6.0%) seropositive samples, 56 corresponded to asymptomatic individuals. According to these large-scale studies, some asymptomatic people showed antibodies, whereas others did not. Therefore, sero-surveillance is limited in the detection of infected individuals.

Slightly high counts were observed in 10 or more samples in our study (specifically, 8, 11, or 20 samples excluding four apparently high samples when cut-off points were 2.0, 1.5, or 1.0, respectively). Most of them showed slightly high results with ELISA or CLIA, although many were below the cut-off value of CLIA. When assayed by two or more methods and slightly positive results obtained concordantly, the detected antibodies might be considered as ones attached to SARS-CoV-2 N antigens. Thus, there are two possibilities: cross-reactivity and low titer of the anti-SARS-CoV-2 N antibody. 

One blood donor sample showed a slightly high value in both RLA and CLIA. Since the sample had been taken more than 10 years ago, it would not be the real antibody against SARS-CoV-2; rather, it might be a cross-reaction of the antibody with a similar antigen. Hence, the slightly high values in some of the hospital staff samples may have been due to cross-reactions. In that case, those having slightly high anti-N titers and normal anti-S titers would not be incompatible.

SARS-CoV and SARS-CoV-2 have similar amino acid sequences (90% similarity, for example, in the N protein) and can cross-react with each other. Most coronaviruses closest to SARS-CoV-2, which are registered in the GenBank database, are detected from bats. Although MERS, human coronavirus HKU1, and human coronavirus OC43 belong to the same beta coronavirus as SARS-CoV-2, they are less similar in sequence (approximately 50.9%, 35.6%, and 36.6%, respectively). Cross-reactions with similar unknown viruses cannot, therefore, be denied. Hörber et al. [9] found slightly high values near the cut-off point in one of three patients with influenza A and two of five patients infected with human coronaviruses. Flink et al. [10] had observed two false positives in two patients infected with human coronavirus OC43; both of them had used S protein as the antigen.

Another possibility for the slightly high results may be the occurrence of very little titer of true antibodies against SARS-CoV-2. In this case, it might be due to either inadequate antibody production and/or lowering of the antibody levels below the cut-off value over time. Long et al. [11] examined 37 asymptomatic patients with COVID-19 along with 37 mild symptomatic ones and found the antibody titers to be significantly lower in the asymptomatic group than in the symptomatic group; the rates of negative conversion after 8 weeks were 40% and 13% in the asymptomatic and symptomatic groups, respectively. Ibarrondo et al. [12] estimated the half reduction time of titers in mildly symptomatic patients to be 26–60 days. Together with the Italian and American large-scale sero-surveillances cited above, these findings indicate that asymptomatic or mildly symptomatic infected people should be included in the group with slightly high values around the cut-off point. Infantino et al. [3] had previously evaluated the iFlashTM CLIA assay system for SARS-CoV-2 antibody employed in this study and reported that the highest sensitivity with a very good specificity performance was reached at a cut-off value of 7.1 rather than 10.0 proposed by the manufacturers. Similarly, Mairesse et al. [4] suggested a cut-off optimization from 10.0 to 4.86 to obtain maximum sensitivities for the same auto analyzing system.

The Ministry of Health, Labor, and Welfare, Japan had conducted sero-surveillance for general citizens in Tokyo, Osaka, and Miyagi Prefectures in June 2020, and reported five out of 2970 Osaka citizens (0.17%) to be positive (https://www.mhlw.go.jp/content/10906000/000640184.pdf, accessed on 30 November 2020). In this study, two commercial kits from Roche and Abbott, both using N protein for antigen, were used. Ten citizens were found positive for Roche’s kit, 16 were found positive for Abbott’s kit, and five were found positive for both the kits. A follow-up study based on neutralizing antibody titration was performed, and only five samples with concordantly positive results had neutralizing antibodies (https://www.mhlw.go.jp/content/000648706.pdf, accessed on 30 November 2020). Since both kits are expected to be highly accurate, samples with high antibody titers cannot possibly be false negatives. Therefore, the discordant samples should have low counts around the cut-off points. Our results regarding neutralizing antibodies being found only in apparently positive samples agreed with the Government’s results.

One of the limitations of our study was the small sample size of COVID-19 patients. More samples from COVID-19 patients could clarify the distribution of the titers of positive samples. Another limitation was that we could not discriminate low-titer true antibodies against SARS-CoV-2 from cross-reactions. This should be clarified in the future. Because RLA is not automated, the lack of accurate procedures might lead to inaccurate quantitation.

## 5. Conclusions

In conclusion, sero-surveillance for SARS-CoV-2 was performed in July 2020 using RLA in a general hospital with a tertiary emergency medical facility for patients with severe COVID-19; only 4 of the 1000 hospital staff (0.4%) tested seropositive, and all 4 had apparent symptoms. Ten or more samples showed slightly high counts, along with slightly high values in CLIA, although they were just below the cut-off point, hence suggesting that the antibody was attached to SARS-CoV-2 N. These results were speculated to imply cross-reactions or low titers of true antibodies against SARS-CoV-2; their accurate evaluation would be useful for epidemiological surveillance in future. Overall, RLA was confirmed to be an excellent quantitative method for detecting very low titers of antibodies.

## Figures and Tables

**Table 1:** Number of positive cases with various cut-off points.

Cut-Off Points (Index)	COVID-19 Patients (*n* = 9)	Hospital Staff (*n* = 1000)	Blood Donors (*n* = 184)
5.0	9 (100%)	4 (0.4%)	0 (0%)
4.0	9 (100%)	5 (0.5%)	0 (0%)
3.0	9 (100%)	6 (0.6%)	0 (0%)
2.0	9 (100%)	12 (1.2%)	0 (0%)
1.5	9 (100%)	15 (1.5%)	1 (0.5%)
1.0	9 (100%)	24 (2.4%)	4 (2.2%)

## Data Availability

Data is contained within the article.
